# The rainbow connection: Disrupting background affect, overcoming barriers and emergent emotional collectives at “Pride in London”

**DOI:** 10.1111/1468-4446.12973

**Published:** 2022-08-29

**Authors:** Chris Robson Day

**Affiliations:** ^1^ Centre for Trust, Peace and Social Relations Coventry University Coventry UK

**Keywords:** collective emotion, LGBTQ+, social justice

## Abstract

This article focuses on a large‐scale parade in the UK that is often overlooked in research concerned with the sociology of political emotions and group dynamics; “Pride in London”. This is an annual parade celebrating, and raising awareness about, the LGBTQ+ community and commemorating the Stonewall riots. Following a brief description of the study context, participants and methods, the article illustrates the use of reflexive thematic analysis of 23 interviewee accounts of the parade. Analysis of emotional habitus and affective practices preceding, and on the day of, the parade offer an insight into the manifestation of collective emotion. Three themes are developed exploring the use of recognizable and emotive symbols, physicality of embodied emotion and spatial arrangement and the encompassing nature of group emotion. Finally, the interplay between background and foreground emotion is explored as a way of understanding and demonstrating the fluidity and temporality of affective experience and expression when people are engaged in collective action at a social justice event.

## 1 PRIDE PARADES

Pride parades are well established events globally where the LGBTQ+ community and their allies can publicly support those who are non‐heteronormative. Often described as commemorations of the 1969 Stonewall riots they have become annual events in many countries and multiple cities (Ammaturo, 2016; InterPride Inc., 2017; Peterson et al., 2018b). These events continue the efforts of the LGBTQ+ community to become recognized, accepted and valued within society. Extensive uptake of the Pride parade format was facilitated by the non‐profit organizations of InterPride, WorldPride, and EuroPride (InterPride Inc., 2017) with the global adoption of the rainbow colors leading to more festive and colorful protests that capture public attention (Peterson et al., 2018a, 2018b; Armstrong & Crage, 2006; Formby, 2017; McFarland Bruce, 2016). While Pride parades continue to become more prevalent, anti‐LGBTQ+ sentiment still exists; in the UK alone, anti‐LGBTQ+ hate crimes in England and Wales having doubled since 2014 (Marsh et al., 2019), public protests against LGBTQ+ ‐inclusive education (BBC News, 2019) and a national survey found a third of the population “remain uncomfortable with same‐sex relationships” (Curtice et al., 2019, p. 130). Resurgence of anti‐LGBTQ+ rhetoric echoes that following the HIV crisis in the early 1980s and the introduction of Section 28; a law banning the promotion of homosexuality in schools, which reinforced negative attitudes toward LGBTQ+ people (Epstein, 2000; Sanders & Spraggs, 1989).

Nevertheless, research consistently surmises that Pride parades promote unity across the LGBTQ+ community (Formby, 2017; Guibernau, 2013; McFarland Bruce, 2016; Peterson et al., 2018b). Additionally, more creative, tactical collective action and visible support have been theorized to result from intensified anti‐LGBTQ+ activities (Stone, 2016). This could indicate why the parade, which was the focus of this research, was attended by 1.5 million people (Pride in London, 2019b, 2019c). Conversely, widespread adoption of Pride and the associated narratives have garnered criticism as a form of homonationalism and colonialism that perpetuate Westernized values of sexuality and gender (Delatolla, 2020; Jackman & Upadhyay, 2014; Rao, 2015). Additionally, the increased popularity and commercialization draws strong criticism for being less radical, more capitalist and evidence that the “LGBT+ psyche has been colonized by a hetero‐normative mentality” (Tatchell, 2019, para. 10; Glass, 2020).

Ceremonies, such as Pride, are social practices which are socially negotiated, emotive, acts of past identity that are performed for oneself and others (Connerton, 1989; de Saint‐Laurent, 2018). Affect and emotion have also been considered as being located in social practices and relations (Gould, 2009; Wetherell, 2012). Consequently, it is the emotional experience of a Pride parade that became the subject of interest for this study to explore the role of emotion at a large‐scale social justice event. Collective emotion is usually conceived as an outcome of the ability to enact identity (Hopkins, et al., 2016; Khan, et al., 2016) through participation in collective action with in‐group members (Drury et al., 2005). Critiques of such research are that group and collective emotion is reduced to an aggregation of scored measures which imply affective experience is a static phenomenon largely experienced in the same way across group members and contingent on a shared identity (Sullivan, 2015; Thonhauser, 2018, 2020; van Kleef & Fischer, 2016; von Scheve & Ismer, 2013). This article will not dispute such conclusions however the findings do suggest they oversimplify role of emotion in groups. Furthermore, this study demonstrated that a Pride parade should be considered a part of an overarching temporal structure rather than as an isolated object of interest (Collins, 2010, 2012; Tilly, 2008).

The article focuses on a parade held in 2019, preceding the COVID pandemic, that was managed by the not‐for‐profit organization “Pride in London” (Pride in London, 2019a). Two conceptual approaches aided exploration of emotional experience at a large‐scale event. Wetherell's (2012) “affective practices”, which situates affect and emotion “in actual bodies and social actors” (p. 159), was considered as the “foreground” where the “intertwining of discourse, affect and practices” (Sullivan, 2015, p. 386) could be investigated in the immediate present. Foreground emotions are often conscious, intentional and temporary phenomena that are situation‐specific and shaped by encompassing, persistent background emotions (Jasper, 2011; Nussbaum, 2001) which act as an affective backdrop helping people navigate the world (Varga & Krueger, 2013). Where an affective practices approach only acknowledges the influence of shared social history (McConville et al., 2014; Wetherell, 2012; Wiesse, 2019) the concept of “emotional habitus” (Gould, 2009) captures how social practices and relations are shaped over time. As the emotional habitus develops so too do possibilities for collective action that are available to a group; predisposing members to particular practices that can be utilized in political action. Importantly, social practice and emotional habitus are interdependent processes which “make, unmake and remake one another” (p. 33) however there is an emphasis on humans as being non‐consciously subjected to affect (Gammerl, 2012).

Investigating these concepts as background and foreground elements in collective emotion provided an affective backdrop to the building of emotion over time (Collins, 2012) and practice of emotion at the parade. Combining the approaches allowed attention to be paid to socially embedded practices that were shaped, but not pre‐determined, by culture. Consequently, novel insights were gained into how social collectives relate to time, disrupt traditional practices and “how collective pasts become sedimented in individual and “collective bodies”, so that the past thus becomes vivified in shared presents” (Narvaez, 2006, p. 52). Furthermore, considering practice and habitus as intertwined ensured the nuance and complexity of emotion and affect was not overlooked (Baker, 2019; Gammerl et al., 2017; Wetherell, 2013) and highlighted how spatial organization and affective arrangements of both physical and psychological space facilitated a dynamic, emotional parade environment (Slaby et al., 2019a, 2019b; Slaby, 2019) rather than serve to mimic social division (Ammaturo, 2016; Johnston, 2007). Finally, the role of allies at Pride is often overlooked in LGBTQ+ research (Formby, 2017; McFarland Bruce, 2016) which means a unique aspect of this study was the investigation of the role of non‐LGBTQ+ allies, their contribution to the experience of (comm)unity and how this can offer an insight into the collective emotional experience of “being moved” (Cova & Deonna, 2014).

### 1.1 Method

Groups participating in the parade, ranging from LGBTQ+ advocacy groups to government institutions, were identified from video footage of the event and contacted by email. A virtual poster asking attendees to share their experience was also circulated on social media. This led to interviews being held with parade participants from different groups and four parade observers; only one of whom was known personally to the interviewer. Transparency regarding my own status as a gay man was essential at this stage in the “ethnographic encounter” (Pink, 2013, p. 37); assisting in trust‐building with gatekeepers or potential interviewees (Gough & Madill, 2012; Plows, 2008; Shaw, 2010) and disrupting heteronormative social expectations (Halkitis, 2015; Roberts, 2014; Schulze, 2015).

Semi‐structured interviews were conducted less than a month after the parade to access relatively recent recollections and ameliorate the unreliability of retrospective memories (Loveday, 2016; Rebstein, 2012). Open questions explored individual and group emotion before, during and after the parade by encouraging self‐reflection and detailed recollections (Kindsiko & Poltimäe, 2019; Potter & Wetherell, 1987; Willig, 2001). Furthermore, group emotions have been demonstrated to be fluid and dynamic in‐the‐moment and over time because they are influenced by contextual factors such as social history and media narratives (Collins, 2012; Hutchison, 2016; Pennebaker & Gonzales, 2009; Ross, 2014; Sullivan & Day, 2019). Consequently, a broader understanding of participant experience was captured by discussing their memories of the weeks and months preceding the parade.

Consistent with Braun and Clarke's (2006; 2019) reflexive thematic analytical approach, verbatim transcripts were created while listening for emotion and documenting the unspoken (Ahmed, 2004; Ayata et al., 2019; Rapley, 2007). The use of “emotion talk” (Edwards, 1999) or conventional emotion labels were limited (Sauerborn, 2019; Wetherell, 2012) therefore initial coding focused on the practice of discursively constructed affective, and affected, bodies, practice and interactions (Berg et al., 2019). Furthermore, group and individual emotions were expected to be indicated by emotional language referencing a wider collective, such as attributions to “we” or “us” or expressed in a manner suggestive of collectively owned emotion (León & Zahavi, 2018; Salmela, 2012; Salmela & Nagatsu, 2016; Tuomela, 2013). Subsequent analysis and theme generation identified shared meaning and diversity of meaning within a topic by demonstrating an intersection between the data and theoretical knowing (Braun & Clarke, 2019).

### 1.2 Recruitment and participants

Nineteen telephone interviews and two in‐person interviews lasted an average of an hour. Two participants answered questions by email. Eight interviewees were lesbian females, four were heterosexual (one of whom was male) and three reported their sexuality as bisexual, polysexual and asexual. None identified as transgender or non‐binary which could be a consequence of self‐selected participation. 50 percent identified as gay, cisgender males, were predominantly White and London residents, however, half reported they were not English (see Table 1).

**TABLE 1:** Participant demographics are as provided by interviewees (only age used preset data that they were required to chose from)

Alias	Country of birth	Gender	Sexuality	Ethnicity	Age range
Alex	UK	Male	Gay	White British	36–45
Anja	Sweden	Female	Heterosexual	White other	46–55
Callum	Scotland	Male	Gay	White Scottish	36–45
Debbie	UK	Female	Lesbian	White British	36–45
Edward	UK	Male	Gay	White British	36–45
Eric	UK	Male	Gay	White British	46–55
Freddy	Mauritius	Male	Gay	Mixed other	26–35
Ginny	England	Female	Bisexual	White British	Under 25
Gordon	Scotland	Male	Gay	White Scottish	36–45
Jordan	UK	Male	Gay	White British	36–45
Karl	Germany	Male	Gay	White European	56–65
Lisa	England	Female	Heterosexual	White British	26–35
Nathan	UK	Male	Gay	Mixed other	26–35
Phoenix	UK	Male	Asexual	White British	Under 25
Quinn	Scotland	Male	Gay	White Scottish	36–45
Rose	UK	Female	Bi/Polysexual	White British	Under 25
Sandra	USA	Female	Lesbian	White American	36–45
Thor	Denmark	Male	Heterosexual	White other	46–55
Vince	UK	Male	Gay	White British	46–55
Warren	Wales	Male	Gay	White Welsh	46–55
Zain	UK	Male	Gay	White British	Under 25
Zara	Malaysia	Female	Lesbian	Malaysian	26–35
Zoe	Greece	Female	Heterosexual	White European	46–55

## 2 ANALYSIS AND DISCUSSION OF THEMES

### 2.1 Emotion‐building before the parade

Emotional temporality is an under‐addressed facet of group emotion (van Kleef & Fischer, 2016; von Scheve & Ismer, 2013) despite individual anticipation of an event having been shown to influence decision‐making and emotional experience (Van Boven & Ashworth, 2007; Wirtz et al., 2003). This became apparent when interviewees were asked about their emotions preceding the parade; the majority of interviewees recounted growing excitement about the parade, for example, Lisa stated that she “was excited. I didn't really know what to expect” while others felt this way because they had enjoyed “Pride in London” previously and “knew kind of what to expect from it which didn't mean that I had less of expectations. Maybe I had even more [laughs]” (Anja). As demonstrated by Sullivan (2018), affective practices in advance of an event play a crucial role in in the formation of individual and collective affective dispositions; here individual feelings were widespread and evoked by a variety of affective practices employed, at multiple levels, in advance of the physical event. Moreover, participants' everyday habitual ways of expressing and enacting emotions about the upcoming parade were different to the everyday fear of the unknown that some interviewees discussed:

There is a huge value in then being together [at Pride] with people whose natural perspective, whose natural understanding of the world, is much more similar to yours… it counters that feeling of isolation that is still all too endemic in LGBT people in general.

(Callum)

Many participants discussed the constant unknown and background emotional habitus for LGBTQ+ individuals where they must be “hyper‐vigilant about whether someone might reject you based on your sexuality” (Zara) and being the only LGBTQ+ person in a workplace, for example, can result in the feeling of “endemic” isolation (Callum). Describing the navigation of heteronormative society as isolating and an unknown, adhering to unspoken rules for LGBTQ+ individuals, is not unfounded; research has found that, for LGBTQ+ individuals, disclosure of their gender or sexuality is a social obligation because failure to do so would be considered deceptive (Billard, 2019; Day & Nicholls, 2019; Lee, 2008; Lee & Kwan, 2014). Other research has identified the cultural pressure to conform to heteronormative ideals through the shaming of queerness that results in social isolation and hiding (McFarland Bruce, 2016). These negative emotional spaces that LGBTQ+ people inhabit on personal and social levels, their emotional habitus, are further exacerbated by broader events. As examples, the mass‐shooting at an LGBTQ+ venue in Orlando (Beckett, 2016), the physical attack on two lesbians on a London bus (Hunte, 2019) and the anti‐LGBTQ+ education protests (BBC News, 2019); these and others were highlighted by interviewees as forefront in their minds. Such events have been found to generate fear for personal safety in LGBTQ+ people (Stults et al., 2017) and add to the collective trauma of historical hate crimes and the HIV/AIDS crisis (Herek, 2017; Nadal, 2018).

Pride‐related activities and practices, in advance of the parade itself, were evident across local‐level, unconnected institutions and accessible to group and non‐group members alike. It mimics the local, micro‐mobilization that often happens in collective action organized by activist social movements (Britt & Heise, 2000; Gould, 2009) and often displays were for the benefit of a more general public:

We have rainbow lanyards which we didn’t have before… our Pride banner up during graduation week on campus to increase visibility. We’ve been flying the Pride flags on our campus during [LGBTQ+] history month.… The build‐up went on for months.

(Warren)

Prior to the physical parade, support for Pride was presented in highly visible ways; wearable items and flags that would be seen by everyone accessing a location and beyond when these displays were shared, positively and negatively, on social media. Individual level efforts, to promote Pride locally, became something workplace collectives endorsed and took ownership of. This is described as Warren talked about the use of the rainbow theme throughout the campus to increase visibility in an ongoing manner. The “we” mentioned refers to the institution rather than a small group, such as an LGBTQ+ staff network. Eric also talked about how “the University actually decorated itself for Pride as well. Which we've never done before. So that was really good”. To clarify, these were not from the same universities, but there were parallel affective practices of endorsement and support being used at other groups and seen in other publicly accessible establishments.

Such displays act to signify the stance of individuals within an establishment and, by default, the institution itself as a collective entity. The emotional journey for some participants begins in advance of the parade as emotions are reified and fostered through the engagement of work colleagues and institutions. The excitement about Pride, generated between colleagues, is amplified and achieves greater resonance with the involvement and approval of their workplace which, in turn, can then lead to more complex and unexpected feelings at an individual level:

I actually got the absolute honour of raising the Pride flag over the hospital this year which was surprisingly emotional. I was a bit taken aback by how emotional.… [in the week before the parade] you looked out across the skyline, and there were Pride flags and one or two Trans flags flying. That is a hugely symbolic thing.

(Callum)

This demonstrates that there is much more than just anticipation or excitement around Pride because it was an event that surpassed the confines of being a physical parade. Actions and activities surrounding it, such as a workplace raising the Pride flag or distributing rainbow‐colored items, encourage feelings of pride, safety, and surprise. The queering of local spaces (Browne & Bakshi, 2011; Doderer, 2011; Peterson et al., 2018b) and the specific social practices participants spoke of are novel and unexpected and act to bolster LGBTQ+ peoples' confidence by alleviating fears or concerns about how they themselves could be received by others.

Flags, particularly those representing countries or cultures, have been identified as totems that are imbued with, and representative of, shared group emotion(s) (Collins, 2004). Such emblems can induce intense feelings, on individual and collective levels, of pride and connection to a social group (Halldorsson, 2020) whilst simultaneously being an immediate symbol of division that can elicit hatred in others (Guibernau, 2013; Moeschberger & Phillips, 2014). The rainbow flag, the Pride symbol, is no different. There are intense positive emotions associated with its presence: namely safety, belonging and trust (Formby, 2017; Wolowic et al., 2017). Such prominent placement of the Pride colors by an institution communicates their institutional norms and values to a wider, public audience. Furthermore, the institution becomes perceived as a space that does not tolerate LGBTQ+ prejudice and therefore a zone of safety.

In contrast to extant research on Pride parades, which often highlights their temporary nature where “for one day a year the streets are queered and the ‘norm’ for the duration of the Pride parade is not heteronormative” (Browne & Bakshi, 2011, p. 181; Taylor, 2014), there is evidence here that, in London at least, the parade was only part of it. Participants described how supportive social practices extended beyond workplaces and localized establishments to the wider world. As evidenced in Callum's description of the city skyline, such practices were clear to see across London. Similarly, Eric recalled that “every government building had a rainbow flag. There's just something brilliant about that… it just felt really good… London felt like it was into Pride this year. There were flags, there were rainbows, there was everything, everywhere”. This queering of space throughout London was recounted by a third of interviewees who mentioned the rainbow colors appearing in shops (Edward), tube stations (Zain) and as pavement decoration (Debbie). Media reports highlighted how national monuments were also utilized, such as Marble Arch being illuminated with the Pride colors, in the week leading up to the parade (Brown, 2019; Buxton, 2019). These affective practices were being enacted collectively at institutional and government levels through symbolic changes to public space throughout London. For LGBTQ+ people this means there are not just supportive institutions but also the city of London that is perceived as a safe, welcoming space which would add to their anticipation and excitement preceding the parade.

#### 2.1.1 Embodied pride and bridging social divides at the parade

Many participants' accounts of the parade day began before arriving at the “holding area” where parade groups waited to start walking. Half of those interviewed spoke about meeting up in small groups, generally the people they would be in the parade with, and getting ready for the day ahead:

We met there [at Regents Park] as a group about an hour and a half beforehand. Some people didn’t know each other so we introduced ourselves. Had some photos. Got excited. Applied more glitter. More makeup. More rainbow colours etcetera.

(Quinn)

Importantly, the majority of interviewees described adorning themselves and each other in this way which indicates such activities formed part of a ritual that acted to bond known and unknown colleagues. Covering themselves in matching, brightly colored, sparkly camouflage brings them together as a cohesive unit through sharing and generating positive feelings together. This is analogous to the interpersonal emotion regulation between soldiers preparing for an exercise or sports teams before a match (Friesen, et al., 2012). Such adornments could also serve to shield them from the gaze of the impending crowds as it offered them a way of “being present/absent by hiding one's personal identity” (Johnston, 2007, p. 47). Accounts suggest a shared social identity or emergent social collective forming; a sense of “we‐ness” and unity without the requirement of pre‐existing social bonds (Drury et al., 2009; Drury et al., 2016; Neville et al., 2020; von Scheve, 2019).

Engaging in this activity with others adds to the excitement and amplifies the flamboyance, inherent to the parade, as emphasized in the way Quinn systematically states “more” before each of the elements. These are the embodied aspects of queer pride that will be seen by everyone. Ritual costuming was not a behavior only adhered to by parade groups; the wearing of Pride colors was highlighted by all interviewees as a conscious activity that began their day whether they were observing the parade or marching in it. As Rose explained, she and her girlfriend were spectators who “met up with my friend [at their house] and we were all getting dressed up. Getting as rainbow as possible. Rainbow make‐up. Rainbow outfit. Rainbow flags and everything” (Rose). The rainbow colors may not be as intensely emotion‐laden for all attendees, particularly those who are non‐LGBTQ+ (Formby, 2017; McFarland Bruce, 2016). However, in advance of and during the parade. They accentuate and create similarity, despite difference, acting as a “cultural bridging practice” (Braunstein et al., 2014).

In a similar way to flags, any objects can become symbols of group membership to which emotional mood are associated. Here, the displacement of attachment onto the Pride rainbow as such a symbol “is a way of steering oneself toward a specific source of emotional energy” (Collins, 2004, p. 317). In this context, of Pride as a special collective occurrence, the adorning of oneself and each other with the rainbow is an example of a widespread, schema‐driven and emotion‐laden socially interactive practice that enhances emotional experience, and commitment to a group, which has been referred to as “ritualized symbolic practice” (Knottnerus, 2010). Taken together, at this point of the Pride event, people were unifying through “getting as rainbow as possible” (Rose), in small groups, over shared meaning(s) of the rainbow flag and, in doing so, they move toward a shared mood. The communal nature of this practice strengthens social bonds and enhances particular emotions within the group (Collins, 2004).

A further important aspect of these prior practices is the flamboyant or “loud” nature of such physical decoration and what it achieves beyond in‐group cohesion. Embodied, visual social practices have been shown to be employed by subcultures with an audience in mind (Feinberg et al., 1992; Tilly & Wood, 2013; Tosoni, 2019). For example, recent research concluded that the Italian goth subculture challenged mainstream conceptualizations of gender, sexuality and beauty using a “strategy of visual shock performed in public spaces” (Tosoni, 2019, p. 37). There is a similar indication in this case study with corresponding goals and performance in a public space, albeit from opposing ends of the color spectrum; thus, rather than emphasizing darkness and unconventional beauty, the Pride parade participants use sequins, glitter, and rainbows. As discussed by Johnston (2007), Pride parades utilize a camp sensibility to challenge heteronormativity through humor and defiance. Donning these colors was about feeling and embodying pride and the transformation of shame (Britt & Heise, 2000); as Freddy described “we felt so proud and beautiful in our NHS t‐shirt, bright colors along with all our colorful glitter and huge Pride flag”. Moreover, the visual display is not necessarily just about confrontation or hubristic display of pride through empowered or emboldened sexuality (Salmela, 2014b) but rather a camp, vibrant manner to encourage inclusion and invite acceptance.

The role of beauty, presenting the LGBTQ+ in ways that would be perceived by onlookers as colorful and attractive, is also highlighted. Where ritualized social practices (Knottnerus, 2010) are emotion‐laden for in‐group members at Pride in London it could be seen that social, or affective, practices were not just for the benefit of the in‐group. Traditionally the LGBTQ+ have been constructed within the heteronormative imaginary as a threat, as deviant and reviled; shared social memory that provides a narrative frame to (dis)empower the collective (Eyerman, 2004; Wertsch, 2009). In this context, the audience is moved to re‐evaluate what they consider beautiful, threatening and, ultimately, acceptable. The bright colors make those in the parade feel a certain way that then also invites onlookers to do the same. An opportunity to disrupt collective memory and negative perceptions by re‐presenting their past identity through the accentuation of queer beauty (Armstrong & Crage, 2006; Johnston, 2007; Narvaez, 2006). The Pride parade becomes the celebration, performance, and embodiment of their past collective, non‐heteronormative identity in the present (Connerton, 1989; de Saint‐Laurent, 2018).

### 2.2 Emotional dynamics in the parade

On the day of “Pride in London”, observers began lining the route a couple of hours beforehand and there was a definite sense of people “settling in” and preparing for the event which would go on for the next few hours. Those who arrived closer to the start time were met with hundreds of thousands of people lining the parade route and had to be prepared to stand with little room for maneuvering between fellow “audience” members. A common feature of Pride parades is that anyone can join and, compared to political protests, “there is a remarkable blurring between the role of marcher and spectator” (McFarland Bruce, 2016, p. 8). This “blurring” is a feature common to many UK and USA Pride parades but not the case at “Pride in London”. Physical barriers separated those in the parade from the co‐present, immediate audience (Abercrombie & Longhurst, 1998). This creates a clear separation between who is, or becomes, the audience and how this is experienced at Pride parades and has been described as a way to “demarcate difference” (Ammaturo, 2016, p. 25). The next section will explore how interviewees engaged with this parade‐observer, or performer‐audience, relationship which served to validate, support, and move, rather than objectify, ridicule and separate, them.

#### 2.2.1 The performance and embodiment of an emerging social collective

Parade elements such as the physical safety barriers have been identified as a hindrance to emotional connection as they transform Pride parades into a stage for identity performance (Ammaturo, 2016). Such measures also constrain the queering of space thereby mimicking the everyday experiences, and emotional habitus, of LGBTQ+ people. Interviewees described the parade itself as a unique, high intensity, and interactive experience where their emotional experience was influenced by, rather than isolated from, the watching crowd. Their accounts indicated that such divisions did not “function as a demarcating instrument which ‘fences off’ the *exoticness* of gay pride participants from the presumably neutral spectators” (p. 25). Recollections were not of an event involving walking through London surrounded by disinterested spectators or feelings of shame as experienced to such a reaction at Edinburgh Pride (Johnston, 2007). Rather, the physical separation and distinct roles at the parade were perceived as a means of overcoming divides between LGBTQ+ and non‐LGBTQ+ groups;

It isn’t just, it’s not stand on the periphery and observe some people doing something. It does feel much more like, although there are physical barriers in terms of railings, it almost didn’t really matter. They just happened to be standing still and we happened to be walking. It didn’t feel like a barrier or a division. It felt like we were all there together.

(Alex)

Barriers were accepted, as part of the event, with the parade and onlookers being understood as being integral parts of that same encompassing entity. The barriers did not prevent Alex feeling that “we were all there together” engaged in the same event just in different ways. He describes a sense of connectedness extending beyond parade group or LGBTQ+ status which was echoed across participants. The sense of togetherness was not simply a presumption inferred from peoples' attendance at Pride, or a sense they had of their own group that they projected beyond the parade, it was informed by observable behavior of the spectators.

Interactions between the parade and the watching crowd featured throughout interviews. There was a sustained collective and “shared emotion of just joy and laughter” (Zara) that was facilitated by the movement of the parade. Whether an observer like Zara, constrained to a fixed location, or in the constantly moving parade, they were one another's highly‐focused, co‐present, immediate audiences (Abercrombie & Longhurst, 1998). As Callum described, there is a symbiotic relationship between the crowd and the parade where “the energy then fuels you and you become much more part of that…the further it went the more that was driving it as well… the more you are playing back to the crowd”. There is a mutual focus of attention on each another and the audience participates in the parade. In line with Collins' (2004) interaction ritual chains there is an exchange of emotional energy that builds over the duration of the parade suggestive of “collective effervescence” (Durkheim, 1912). What is further hinted at, which is not well explored by Collins or Durkheim, is the intentionality, the cognitive aspect, within collective effervescence or collective emotion (Salmela, 2014a; von Scheve, 2011). This is not a passive acknowledgment of others being involved, and experiencing similar feelings, there is an active, deliberate engagement on both sides; “playing back to the crowd” describes a response, rather than deliberate and unsolicited action to incite reaction with the use of “playing” denoting it as pleasurable. Similar collaborative and playful activities were recollected by most interviewees as observable in a variety of crowd actions, such as cheering, screaming and laughter, which were recalled as affiliative, affective behaviors.

Within this, the watching crowd is also an audience for the parade and, despite there being little research on audience emotions as a group (Kolesch & Knoblauch, 2019), there are collective modes of emotional expression in operation. These are not simply “a ‘reaction’ to what is happening, they also *perform and reflect* specific situational entanglements and communicate as embodied evaluation of them” (p. 256). The reciprocal nature of this emotional behavior was not restricted to observation and vocal interaction; there was also a physicality that defied the separation of the safety barriers. As the parade progressed “it started, in a way, to feel like a street party… [the crowd] were standing and cheering but now they're actually reaching out to engage [with those in the parade]” (Edward). Surprise that this happened was indicated by his tone and the emphasis on “actually”. Nearly half of the interviewees referred to such physical engagement in a similar way. For example, Alex echoed this when he said, “they actually do, they want to reach in and high five you” and Zain described how the watching crowd “wanted to give you a kiss. They wanted to give you a hug”. The salience of physical contact may be linked to LGBTQ+ individuals having personally experienced societal aversion to touching them or being near them throughout their lives (Froyum, 2007; Holmes & Cahill, 2004). Social revulsion in the form of overt prejudice and covert microaggressions are similar shared experiences, at school or work for example, and form part of their collective memories (Nadal, 2018; Wertsch, 2009). Institutional anti‐LGBTQ+ narratives and the construction of HIV as the “gay plague” fueled fears of casual transmission from contact with LGBTQ+ individuals (Chubb & Fouché, 2020; Flowers & Langdridge, 2007; Gould, 2009; Lerche, 2016; Swain, 2005).

Emphasis was placed on how spectators chose to initiate, and make a conscious effort, to make physical contact. Boundaries were blurred between the audience, as being a passive receiver or observer, and the expressive crowd (Abercrombie & Longhurst, 1998; Kolesch & Knoblauch, 2019). At this event, they are an *expressive audience* who desire connection with the people they are watching; these physical interactions bring the audience into the performance of the parade. The need to physically connect with those in the parade evidences the role of touch in the sharing of experience. Haptic sensation is often overlooked in favor of facial‐visual sharing even though it has been connected to how human experience begins in utero and the world is a shared experience physically through their mothers (Ciaunica, 2019). For recipients, it demonstrated a more unconditional support, reminiscent of loving behaviors, and reassurance that the audience were not solely there as impassive voyeurs or consumers of a performance.

#### 2.2.2 Affectively moved by others

Another emotional response that deserves exploration in this situation is the simultaneous experience of positive and negative feelings that interviewees presented as unexpected. It is a state that has been described as: “elevation” (Haidt, 2000, 2003) in response to witnessing examples of moral beauty or human virtue (Thomson & Siegel, 2017), “being moved” by situations where “positive values are brought to the fore and manifest themselves in a particularly salient way” (Cova & Deonna, 2014, p. 8; Cullhed, 2020) or “kama muta”, an encompassing experience found across time, language and culture, to a wide variety of stimuli; from cute images of animals and babies to instances of union and oneness with others (Fiske, 2019; Fiske et al., 2017). Despite considerable variation, the literature agrees that it is a positive emotional experience, with an element of sadness, and that physiological responses can include a warmth, or tingling, in one's chest, suppressed or shed tears and piloerection (goosebumps) (Zickfeld et al., 2019). Further, experiencing this emotion results in feeling more connected to, or positive about, others and increases helping behaviors (Thomson & Siegel, 2017; Zickfeld et al., 2019). Studies have shown it to be a low affective arousal, but high intensity, emotion (Menninghaus, et al., 2015) that, unlike nostalgia, does not involve a longing for the past and is “an other‐oriented emotion, [whereas] nostalgia is primarily categorized as a self‐relevant emotion” (Zickfeld et al., 2019, p. 126).

The feeling of being moved or touched, as an individual, was recounted by some interviewees as a response to the appearance of Pride colors across London in the lead up to the parade although the main associations made were to visibility, relief, and safety. Similarly, emotions and emotional reactions, of themselves and others while in the parade, were described throughout the interviews supporting claims that this was a space of safety and relief:

Some people are even in tears. As you might know yourself, if you’ve been on these marches, it’s very moving… I guess it was excitement? Pride? I remember feeling emotional. I remember feeling a bit upset. I say upset but upset is not the right emotion. But I was ready for kind of crying but I think it was the idea of being able to do that without fear of judgement? Probably the relief that that brings.

(Quinn)

When describing how others around him were reacting Quinn reflected on how this resonated with his own emotions being in the parade. He began by focusing on the main positive emotions he remembered but quickly concludes they are insufficient as an explanation. The apparent confusion and questioning involved in his response could reflect difficulty navigating this positive experience because he realizes that he, and therefore others, were not just experiencing joy.

Although relief was discussed by the majority of LGBTQ+ interviewees there were also those who were non‐LGBTQ+ attending Pride in London. Zoe, a heterosexual female from the Mediterranean, recollected that her feelings in the parade “was good overwhelming feeling… I had the experience to be somewhere that homosexuality is accepted and celebrated… I would have cried. Tears would come in my eyes… it was a happy emotional state”. Once again, the description is of being moved to tears and this feeling was associated with a variety of positive emotions. This was echoed by Thor, a Scandinavian, heterosexual male who described feeling “totally blown away… Literally tears in my eyes when I was walking… it was so touching and moving with all the love and happiness”. There were similarities between these two individuals as non‐UK natives, attending their first Pride parade and not identifying as LGBTQ+. Conversely, their life experiences differed in terms of gender and, as per their own accounts, because Thor was socialized in a progressive country and Zoe in one with conservative values. Despite this, their affective experiences resonated with those of LGBTQ+ interviewees.

The connection between these intense emotions can be appreciated through consideration of where they are consistent. First is the desire to cry, which was powerfully felt but not enacted. While tears, in adults, are generally perceived as a social cue of distress or sadness they can also be felt, and shed, in response to overwhelmingly positive situations when behavioral control is relinquished (Gracanin et al., 2018). As a celebratory event, in which interviewees were surrounded by strangers, it would be inappropriate and uncomfortable, for themselves and others, to appear vulnerable or requiring help (Gammerl et al., 2017) and physical tears can elicit negative emotions from others (Hendriks et al., 2008). Accounts demonstrated a conscious effort to control the expression of emotion (Hochschild, 1979) that could be perceived, incorrectly, as negative by those around them. Alternatively, an inability to make sense of such an erroneous reaction, in the moment, leads to it being interpreted, and expressed, as something else (Fiske et al., 2017).

Secondly, there was a difficulty expressing exactly what emotions or affects were felt. Quinn poses alternative emotions as questions while the majority of interviewees described their experience as positive but “overwhelming” or “overpowering”. Demonstrated in these emotional combinations, is the emotion of “being moved” (Cova & Deonna, 2014). There is an intensity to these accounts that goes beyond that conceptualized in “kama muta” (Fiske, 2019; Fiske et al., 2017) and not restricted to the observing of moral beauty or virtue as with “elevation” (Haidt, 2000, 2003). Awe is a possible alternative description but this is generally associated with reverence at being confronted by power or vastness (Keltner & Haidt, 2003; Menninghaus, et al., 2015; Zickfeld et al., 2019) however interviewees did not hint at a perception of overwhelming power or a desire to submit to one. Participant accounts emphasize the observable positive emotions, and values, in others; locating their emotional responses in, or with, them and providing descriptions that resonate with the concept of “being moved” as an “invasive depth of the feeling […] but also the concomitant sense of satisfaction or contentment, a state of relief perhaps” (Cova & Deonna, 2014, p. 12).

“Being moved” has been theoretically applied to groups and collectives; “kama muta” was proposed as a more psychologically robust theory to explicate collective effervescence (Durkheim, 1912) and Turner's (1969) concept of communitas (Fiske et al., 2019). Similarly, “being moved” as a collective has often been considered in terms of the emotional motivators that mobilize a group to collective action (Eyerman, 2005; Gollnhofer & Kuruoglu, 2018). Laboratory research found the feeling of “being moved” was less intense when imagined in a public setting (Jakobs et al., 1996; Manstead & Fischer, 2001) which suggests its inhibition rather than amplification in a social setting. Contrary to this, findings indicated that being moved emotionally was not inhibited in the presence of others and that it could also be experienced as a result of collective action. Rather, the collective plays a role in shaping and understanding this emotion. In a group setting there may be no conscious sharing of a potentially private, individual affective state of being, however, it is recognized in co‐present others and can become synchronous. Finally, this emotion could not be directly tied to a shared identity because it was experienced by LGBTQ+ and non‐LGBTQ+ individuals alike from diverse backgrounds and organizations. Interviewees made a connection with these feelings and the embodiment of emotion in others rather than associating them directly with personal feelings of pride or safety for example, It complements the extant literature by demonstrating an emergent social collective or shared social identity through shared collective experience (Mühlhoff, 2019; Neville et al., 2020; Salmela, 2012; von Scheve, 2019).

### 2.3 General discussion

As discussed, beyond the physical event of the parade there were affect‐laden practices employed at multiple levels of society; social and political movement‐style tactics of local, micro‐mobilization were used in conjunction with an implicit style of advertising and promotion. Leading up to the parade the London cityscape was gradually queered over time as supportive businesses and institutions displayed Pride colors in various forms. Such encompassing approaches are not common practice for all UK Pride events which can result in them being public events that are poorly received (Johnston, 2007). As societal affective practices they were communicating with an imagined audience comprised of supporters and non‐supporters. This meant that there was a shift in emotional habitus for many LGBTQ+ interviewees who associated this queering of space with more than just feelings of pride; the city was signaling that they were welcome and would be safe. Simultaneously, these actions acted to “prime” the general public for the upcoming event by preparing the collective for a weekend when London would become an inclusive space. While individuals and groups may have had some influence in these shows of allegiance, in their own workplace for example, this aspect in the build‐up to the event was beyond the direct control of most participants. The pre‐configuration of public space by bastions of authority, such as “Pride in London” organizers and government officials, heralded that Pride, and by default the LGBTQ+ community, was an unquestionable and integral part of London. Moreover, the prominence of rainbow colors and flags in physical spaces constructed and positioned the LGBTQ+ in the collective imaginary as being “ours” and promoting a sense of togetherness in the capital city.

All interviewees described the atmosphere of “Pride in London”, not just their individual emotions or those they felt on behalf of a group, but rather their perceptions and accounts of affect around them before and during the parade. The extension of positive feeling beyond the physical parade was contingent on collective affective practices that preceded the event combined with feelings and observations on the day itself. Pride colors were an immediately recognizable affective practice of support and similarity that signified shared affiliations serving to connect attendees and fostering a sense of belonging, inclusion, and togetherness (Formby, 2017; Guibernau, 2013). As with sports merchandize, this creates and affirms individual and collective identity to “promote a sense of community among supporters and facilitate social integration” (Derbaix & Decrop, 2011, p. 288) as well as encourage inclusivity because they could be worn or displayed by anyone (Stieler & Germelmann, 2016; Thonhauser & Wetzels, 2019). The sense of togetherness, within and beyond the parade, would be reinforced by the shared Pride symbols and blurring of boundaries between us‐and‐them that this achieves. Everyone wearing Pride colors presents and performs a non‐heteronormative collective identity where sexuality becomes hidden and cannot be presumed; a direct contrast to current societal organization where there is an expectation of being heterosexual and cisgender (Brown et al., 2016; Browne, 2007; Formby, 2017). Past LGBTQ+ identity is embodied, reinforced, and extended to others in the present to (re)imagine the future (Connerton, 1989; de Saint‐Laurent, 2018; Narvaez, 2006).

Contrary to previous research on Pride parades (Ammaturo, 2016; Johnston, 2007), the physical setup and separation of the event was not found to reinforce social divisions or hamper feelings of liberation. Instead, they encouraged interactions between observers and those in the parade. This collaborative performance was a source of feelings of togetherness and shared emotion. Observers reaching in to touch those in the parade was a source of surprise linked to the LGBTQ+ collective memory and emotional habitus. The surprise that such simple social interactions elicited, initiated by the observing crowd, highlights an underlying, implicit or unacknowledged shame that was a component of interviewees' emotional habitus. Remarkably, the interactions that were understood as signifying a form of embodied support and togetherness were *facilitated by* the barriers separating those in the parade from the spectators. In a freer spatial arrangement, without the performer‐observer dynamic, there would have been no requirement to reach out and connect in such a way. While this would have meant the parade was a less restricted queering of space, where people were not performing their identity for an audience, participants would have been prevented from engaging in such deliberate, unity‐evoking affective encounters where subject and object were connected in a manner that subverted social and societal power relations (Butler, 2015; Papenburg & Zarzycka, 2013).

For individuals who experienced such affective encounters these were often remembered as personally significant even though they were not instances of a collective behavior, experience, or response. Rather, the unexpected, collective practices of support, from the presumed out‐group, before and during the event led to an expanded sense of unity that left interviewees feeling moved and positively overwhelmed. Pervasive as this powerful emotion was, “being moved” was an unfamiliar state which people did not consciously share with others and unlikely to have been identified without the depth afforded from interviews. Potentially interviewees made sense of the affective experience as personal because they were unsure how to express, interpret or make sense of a mixture of tears and happiness in such a positive environment. Emotions recounted are those that are perceived in, or are a response to, collective emotions of others and, as shown in the analysis on “being moved”, affect is perceived as tangible and inspires sharing of emotions and a sense of resonance, or togetherness, between people. As a response to the collective behavior and emotions observed in others being moved supports the notion of unexpected features of joint affective practices heightening collective emotion and being more memorable. Emotional habitus explanations emphasize the involuntary, non‐conscious properties of affective encounters (Gould, 2009) whereas the findings of this study would suggest the impact of collective agency where background affect can inform and constrain, but not prescribe, the social practice of emotions and the possible influence of innovative affective practices in the present (Gammerl, 2012; Wetherell, 2012).

This research found that social collectives can be moved emotionally when they are physically engaged in the same form of co‐present collective action. Being moved is not being considered as an embodied affective practice to *consciously* communicate feeling, however, it was recognized as a unique felt affective response in oneself which resonated with others. Crucially, it was not an uncommon experience and was recounted by various interviewees with no prior connections. As it was independent of organization, social group membership and specific location it suggests the feelings were widespread and genuinely collective. Moreover, as in Quinn's account, it was an emotional state recognized in others at the parade. It is appropriate to consider this as a manifestation of collective emotion(s) because collective emotions are not solely predicated on a defined shared identity, rather, the parade fulfills the elements of co‐presence, co‐ordinated behavior based on collective concerns and goals for the event (Sullivan, 2015; Thonhauser, 2020; von Scheve & Ismer, 2013). Considering these elements in tandem with participant recollections indicates synchronicity and convergence of “being moved” emotionally as a collective.

Finally, the LGBTQ+ community is an amalgamation of identities rather than a single, encompassing social identity; there are, of course, potential overlaps however it is important to remember they are not all one and the same (Formby, 2017; Ghaziani, 2011). For some parade attendees the positive emotions were a consequence of being able to enact individual identities, but it would be disingenuous to state this was the case for the majority. Participants in this study do not represent the diversity and complexity of LGBTQ+ people because there was insufficient participant variation in terms of cultural background, gender‐identity, ableness and education. Where variation in perspectives was achieved was along “traditional” demographics such as age, gender, and sexuality which is a limitation of the study that requires attention in future research. The novel inclusion of ally accounts demonstrated that even though they did not have comparable social histories they recounted an event as intensely positive and unifying as those who identified as LGBTQ+. Across a multiplicity of identities attending the Pride parade there were collaborative, joint behaviors and responses that generated collective emotion making this Pride parade a consensual performance and sedimentation of an existing identity as well as the extension of that identity to include others beyond the in‐group.

## 3 CONCLUSION

This case study of “Pride in London” demonstrates how Pride parades are spaces where people can come together to experience positive emotions with others, enact personal and shared identities, and foster social ties and feelings of togetherness. The parade itself is demonstrated to be an affective experience that extends beyond socially constructed identities and the geographical and temporal restrictions of the physical parade. Culture, shared knowledge, and collective memory were found to provide background and context to the social practices of affect that were employed in the present. Emotional habitus and affective practices before the parade fed into the building of emotion over time. Spatial organization and affective arrangements of both physical and psychological space facilitated a dynamic emotional parade environment. Subsequently, the joint affective practices, shared experience and collective emotion transcended identification with social groups. The emergence of collaborative emotional behaviors in‐the‐moment (i.e., at the event) were dependent on the confluence of background emotions, the affective backdrop, and the physical and tangible affect encountered on the day. Being cognizant of the interdependent foreground and background relationship of affect and emotion is essential in the planning and management of large, public gatherings to increase the engagement and enjoyment of attendees.

## CONFLICT OF INTEREST

There are no conflict of interest identified by the author.

## Data Availability

The data that support the findings of this study are available from the corresponding author upon reasonable request.
